# Scientific Misconduct and Social Media: Role of Twitter in the Stimulus Triggered Acquisition of Pluripotency Cells Scandal

**DOI:** 10.2196/jmir.6706

**Published:** 2017-02-28

**Authors:** Yuya Sugawara, Tetsuya Tanimoto, Shoko Miyagawa, Masayasu Murakami, Atsushi Tsuya, Atsushi Tanaka, Masahiro Kami, Hiroto Narimatsu

**Affiliations:** ^1^ Institute for Promotion of Medical Science Research Yamagata University Faculty of Medicine Yamagata Japan; ^2^ Department of Health Policy Science Graduate School of Medical Science Yamagata University Yamagata Japan; ^3^ Cancer Prevention and Control Division Kanagawa Cancer Center Research Institute Yokohama Japan; ^4^ Navitas Clinic Tokyo Japan; ^5^ Faculty of Nursing and Medical Care Keio University Fujisawa Japan; ^6^ Graduate School of Science and Engineering Yamagata University Yonezawa Japan; ^7^ Medical Governance Research Institute Tokyo Japan; ^8^ Department of Public Health Graduate School of Medical Science Yamagata University Yamagata Japan

**Keywords:** web 2.0, bioethics, Internet, mass media

## Abstract

**Background:**

The academic scandal on a study on stimulus‑triggered acquisition of pluripotency (STAP) cells in Japan in 2014 involved suspicions of scientific misconduct by the lead author of the study after the paper had been reviewed on a peer‑review website. This study investigated the discussions on STAP cells on Twitter and content of newspaper articles in an attempt to assess the role of social compared with traditional media in scientific peer review.

**Objective:**

This study examined Twitter utilization in scientific peer review on STAP cells misconduct.

**Methods:**

Searches for tweets and newspaper articles containing the term “STAP cells” were carried out through Twitter’s search engine and Nikkei Telecom database, respectively. The search period was from January 1 to July 1, 2014. The nouns appearing in the “top tweets” and newspaper articles were extracted through a morphological analysis, and their frequency of appearance and changes over time were investigated.

**Results:**

The total numbers of top tweets and newspaper articles containing the term were 134,958 and 1646, respectively. Negative words concerning STAP cells began to appear on Twitter by February 9-15, 2014, or 3 weeks after Obokata presented a paper on STAP cells. The number of negative words in newspaper articles gradually increased beginning in the week of March 12-18, 2014. A total of 1000 tweets were randomly selected, and they were found to contain STAP-related opinions (43.3%, 433/1000), links to news sites and other sources (41.4%, 414/1000), false scientific or medical claims (8.9%, 89/1000), and topics unrelated to STAP (6.4%, 64/1000).

**Conclusions:**

The discussion on scientific misconduct during the STAP cells scandal took place at an earlier stage on Twitter than in newspapers, a traditional medium.

## Introduction

In recent years, the number of cases of scientific misconduct, including fabrication, falsification, and plagiarism has increased [1]. Misconduct damages scientific progress and public trust; in addition, the ensuing incorrect research results threaten people’s health [2]. As of May 2012, 2047 papers in the fields of medicine, biology, and life science were retracted from PubMed, a fully accessible database on biomedical literature. The reasons for the retraction were error (21.3%), fraud or suspected fraud (43.4%), duplicate publication (14.2%), and plagiarism (9.8%) [3,4]. Japan had the third largest number of retracted papers due to fraud or suspected fraud in the world [5].

The three major historical scandals of scientific misconduct were German physicist Jan Hendrik Schön’s fraudulent superconductor breakthroughs at Bell Labs in the United States in 2002, South Korean researcher Hwang Useok’s fabrication of embryonic stem cells in 2005, and Japanese stem-cell biologist Haruko Obokata’s and her fellow researchers’ claims on stimulus‑triggered acquisition of pluripotency (STAP) cells in 2014 [6,7]. Suspicions on the three studies were raised in different arenas. Researchers who were unable to replicate Schön’s results raised their concerns in a conventional reseacher’s community [8], whereas discussions and debates on Useok’s study occurred on Korean and Japanese Web-based message boards, respectively [9]. The allegations of misconduct of Obokata, who led the STAP cells study, spread on Twitter after their paper had been publicly reviewed on PubPeer [10,11]. The hurling of accusations worldwide on a single paper by a large number of Twitter users, including many nonspecialist members of the public, attracted widespread attention. Their misconducts included “copying and pasting,” which were familiar methods to the public; thus, the public could join the discussion.

The STAP cells scandal demonstrated how Twitter enables the rapid spread of information through sharing between multiple users, allowing numerous users to obtain the information simultaneously. Suspicions on research papers, thus, can be raised on Twitter by multiple people, making the social media site a useful tool for discussion and debate. In fact, previous studies have found that researchers engage in discussions on their studies via Twitter [12,13]. Several studies have examined the concern expressed by the chief editor of a scientific journal regarding the self-plagiarism conducted by a chemist [14] and the controversy regarding Felisa Wolfe-Simon’s claims on Twitter about the bacteria that lived without phosphorous [15]. Through Twitter, a rapid response to questions on misconduct through a debate is possible and discussions lead to an exchange of a diverse range of opinions. Such processes generate more questions and play a role in dispute resolution. However, a widespread controversy on Twitter may have a restraining effect on the concerned researchers, and Twitter’s roles as a tool for discussion and later dispute resolution are yet to be determined.

This study investigated discussions on STAP cells on Twitter and the content of newspaper articles in an attempt to differentiate social from traditional media. To identify changes in the tone of Twitter and newspapers over time, collected tweets and newspaper articles on STAP were decomposed through a morphological analysis. Furthermore, to ascertain social media’s role in resolving scientific misconduct, Twitter’s role in the STAP cells scandal was compared with that of newspapers, which represent the traditional media.

## Methods

### Collection of HTML Files

Searches for tweets and newspaper articles containing the term “STAP cells” were carried out for 6 months (26 weeks) from January 1 to July 1, 2014. Tweets were extracted through Twitter’s search engine. A top tweets search was performed every week; top tweets are tweets that have been retweeted or replied to by several users and selected through an algorithm developed by Twitter [16]. The newspaper article search covered Japan’s 5 major national newspapers (*Asahi Shimbun*, *Yomiuri Shimbun*, *Mainichi Shimbun*, *Sankei Shimbun*, and *Nihon Keizai Shimbun*). The 5 major national newspapers published a combined total of 23,543 thousand copies every half-year on average [17,18]. Articles were extracted from Nikkei Telecom, a Japanese newspaper article database [19].

Tweets and newspaper articles generated from the search were saved as HTML files by using the Web browser function. One HTML file contained a week worth of search, except from April 9 to 15, when the number of tweets was so high that weekly data could not be saved in one file. As such, HTML data were saved separately for each day. The syntax for designating cut‑off dates provided on each of the official sites was used in each search.

### Extraction of Japanese Text Data

Text data were extracted by stripping the HTML tags from the HTML files saved from the Web browser. The dates, account names, text of the tweets, and the titles and text of newspaper articles were extracted from the Twitter HTML and newspaper article files, respectively. Nadeshiko (a Japanese programming software, Free edition, Kujirahando, Japan) was used to write a program that would eliminate tags and extract the text.

### Text Processing

To generate the relevant words for the morphological analysis, the dates, account names, URLs, and graphic characters were eliminated from the tweet text data. The following frequently used terms were also eliminated from both tweets and newspaper articles: STAP, stap, cells, Obokata, Haruko, RIKEN Research Center, and RIKEN. The free bulk text processing software Text Search and Substitute.NET (TextSS.NET) version 5.21 (Yamashita-Y, Japan) was used for text processing.

### Morphological Analysis

Morphological analysis is one of the basic techniques used in Japanese text mining analysis. It is the process of segmenting a given sentence into a row of morphemes. A morpheme is a minimal grammatical unit, such as a word or a suffix [20]. MeCab (Graduate School of Informatics Kyoto University, Kyoto, Japan, and NTT Communication Science Laboratories, Seika, Kyoto) performs morphological analysis using the hidden Markov model and statistical processing [21].

Morphological analysis of tweets and newspaper articles was performed for each week for 14 weeks (3 months and 1 week, from January 22 to April 29), covering the week before the publication of the STAP cell paper (Week 0) and the 13 weeks after (Weeks 1-13). The nouns identified in the tweets and newspaper articles through the analysis were extracted, and their frequencies were calculated.

The top 100 nouns that appeared most frequently each week in tweets and newspaper articles during the 14 weeks were then extracted. Terms that were either positive or negative toward STAP cells were then selected from among these 100 frequently appearing nouns. Changes in the use of these positive and negative terms each week were also investigated. Authors Yuya Sugawara, a specialist in medical informatics, and Hiroto Narimatsu, a medical doctor, selected the positive or negative Japanese terms semantically.

R version 2.13.0 (R Foundation for Statistical Computing) was used for morphological analysis. The R package used for our morphological analysis was RMeCab version 0.97. RMeCab is a package developed to operate the Japanese morphological analyzer MeCab version 0.98 from R.

### Extraction of Accounts and Tweets

To investigate the attributes of Twitter account holders who tweeted using negative terms on STAP cells, 100 from the total 558 accounts that sent STAP‑cells tweets from February 5 to 18, 2014, containing the negative term “unnatural” were randomly selected. Profile searches were performed on these 100 accounts using Twitter’s Profile Search feature [22]. All the 558 accounts were assigned random numbers. We extracted 100 accounts in the descending order of the assigned random numbers using Excel (Microsoft Corporation, Redmond).

In order to classify the content of tweets on STAP cells, 1000 tweets were randomly extracted from the total 134,958 tweets that were assembled over the 6-month search period; all the 134,958 tweets were assigned random numbers, and the 1000 tweets were extracted in the descending order of the assigned numbers. These 1000 tweets were classified into four types: STAP related; links to news sites, and so on; false scientific or medical claims; and topics unrelated to STAP specified by Yuya Sugawara and Hiroto Narimatsu.

## Results

### Number of Newspaper Articles and Tweets Per Week

**Figure 1:**
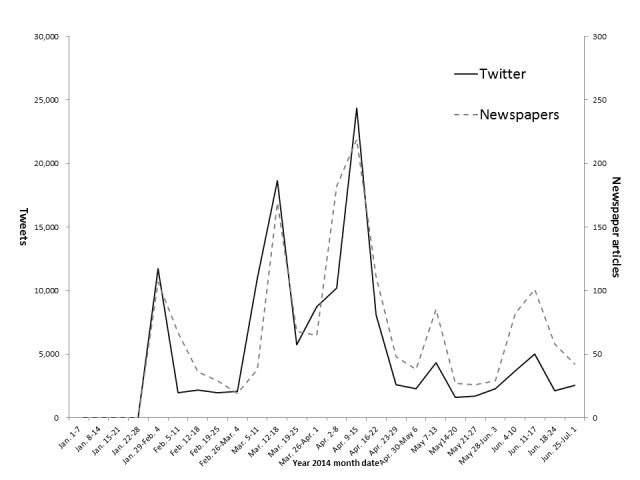
A comparison of the numbers of tweets and newspaper articles containing the word “STAP” between January 1 and July 1, 2014. The solid line shows the number of tweets and the broken line newspaper articles.

Figure 1 shows the numbers of newspaper articles and tweets containing the term “STAP cells” each week for the 6-month period following the publication of the STAP cells paper. A total of 1646 newspaper articles and 134,958 tweets appeared in 6 months. The number of both newspaper articles and tweets followed a similar trend, exhibiting transient increases during the periods from January 29 to February 4, from March 12 to 18, and from April 9 to 15. The numbers of tweets and newspaper articles were correspondingly 11,718 and 107 during the first period, 18,649 and 169 in the second period, and 24,344 and 219 in the third period.

### Timeline of the STAP Cells Scandal

**Figure 2:**
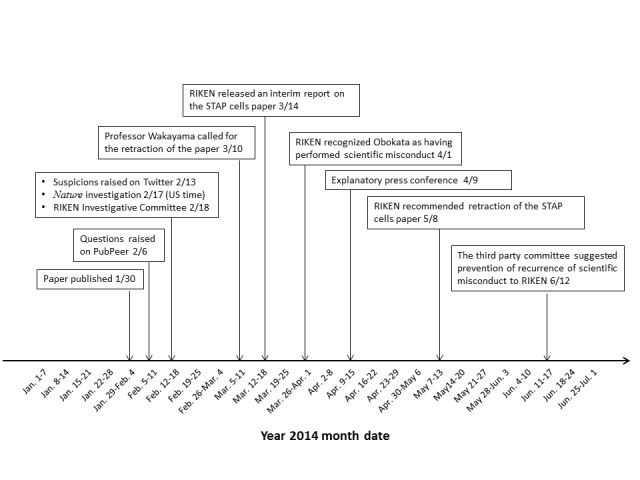
Timeline of events related to the stimulus triggered acquisition of pluripotency (STAP) cells scandal.

Figure 2 shows the timeline of the STAP cells scandal. The paper on STAP cells was published on January 30, 2014 [23]. On March 14, RIKEN released an interim report on the paper [24]. Subsequently, on April 9, Haruko Obokata held a news conference [25].

### Frequency of Nouns Appearing in Tweets and Newspaper Articles

We conducted a morphological analysis of the tweets and newspaper articles that appeared during the first 13 weeks (3 months) following the publication of STAP cells paper on January 30. The frequency of appearance of nouns in these tweets and newspaper articles during the same period was also investigated. A total of 100 nouns that appeared most frequently were extracted and classified whether positive or negative toward STAP cells. The positive terms selected were “major discovery” and “ground-breaking,” whereas the negative terms were “unnatural,” “fabrication,” and “falsification.”

**Figure 3:**
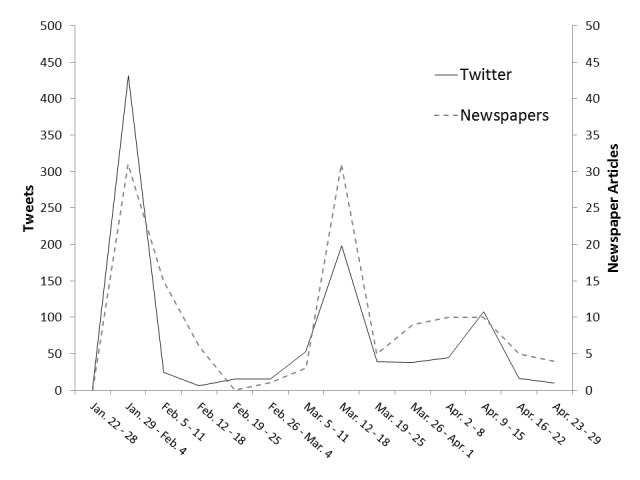
Frequencies of the use of positive terms related to stimulus triggered acquisition of pluripotency (STAP) in Twitter and newspapers.

**Figure 4:**
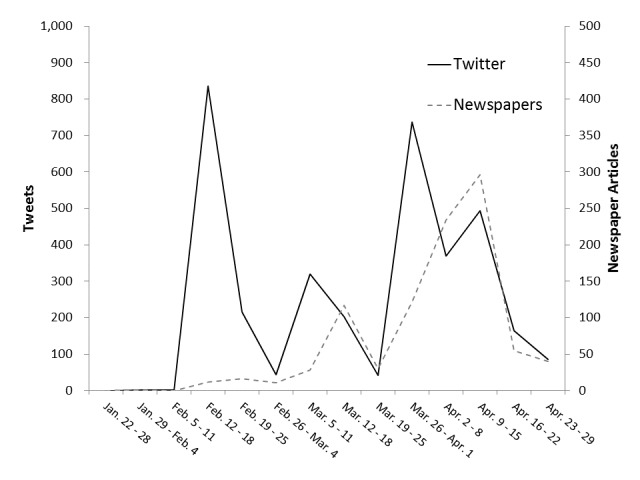
Frequencies of the use of negative terms related to stimulus triggered acquisition of pluripotency (STAP) in Twitter and newspapers.

Figures 3 and 4 show the frequency of use of positive and negative terms in tweets and newspaper articles during the first 3 months (13 weeks), including 1 week before paper publication (14 weeks in total), following the publication of the STAP cells study. In Twitter, the frequency of positive terms was the highest (n=432) in the period from January 29 to February 4, whereas the frequency of negative terms was the highest (n=835) in the period from February 12 to 18. The frequency of negative terms was 1.93 times higher than that of positive terms. In newspaper articles, the frequency of positive terms was the highest (n=31) from January 29 to February 4 and March 12 to 18, whereas the frequency of negative terms was at the maximum (n=296) from April 9 to 15. The frequency of negative terms increased 6 weeks after the STAP paper publication. The highest frequency of negative terms in newspaper articles appeared 8 weeks later compared with that for tweets.

### Account Attributes and Tweet Content

Four of the account holders who published tweets on STAP cells containing the negative term “unnatural” were considered to possess certain specialist knowledge being a former biological researcher, a staff at a manufacturer of reagents for biological experiments, a science writer, and a science journalist. The others included 8 news sites, 9 bots (accounts that generate tweets automatically), and 79 accounts for which the level of specialist knowledge could not be determined.

Of the total 134,958 tweets generated during the 6-month period, 1000 tweets were randomly selected and found to contain STAP­-related opinions (43.3%, 433/1000), links to news sites and other sources (41.4%, 414/1000), false scientific or medical claims (8.9%, 89/1000), and topics unrelated to STAP (6.4%, 64/1000). The examples of tweets that were evaluated to make false scientific or medical claims were as follows: “STAP cells will give us immortality,” “STAP cells are produced from the skin of newborn babies,” and “STAP cells can be made at home.”

## Discussion

### Principal Findings

This study revealed that the discussion on misconduct in the STAP cells affair was taken up at an earlier stage on Twitter than by newspapers. Positive terms appeared both on Twitter and in newspaper articles during the first week after STAP cells study was published, suggesting that the tendency was for STAP cells to be viewed positively. From Week 2 (February 5-11), however, the use of positive terms decreased and negative ones started to appear. On the Twitter, the frequency of negative terms increased during February 12-18 and starting again from March 19 to 25, 2 weeks after Obokata’s coauthor called for the retraction of the paper. In newspaper articles, the negative terms increased between March 19 and 25 and April 9 and 15; an explanatory press conference by Obokata was held during April 9 and 15. The highest use of negative terms in newspaper articles was observed during this week. The use of both positive and negative terms declined beginning during April 16-22. The story on suspicions were taken up by newspapers after the “unnatural” nature of STAP cells had been pointed out on Twitter and after RIKEN announced that it had found Obokata guilty of scientific misconduct, with the increased use of “fabrication” and “falsification” setting a different tone. This tone might have affected public opinion on the STAP cells paper. In both the Schön and Useok scandals, both of which constituted serious scientific misconduct, the retraction of papers took several years [26,27]. Obokata’s STAP cell paper, however, was withdrawn after only about 5 months [23,28]. New tools such as Twitter might have played a role in the early process leading to the retraction of the paper in the STAP cell scandal. Publishing on Twitter has the clear advantage that it is speedy [14]. The greatest advantage of using Twitter for scientific discussion is the rapid result of a debate. Scientific misconduct has continuously occurred after the Useok scandals. In Japan, Valsartan-related misconduct occurred [5,29-33], and after its publication in 2007 [34], a researcher expressed concerns about the study [35]. The misconduct by an employee of a pharmaceutical company was revealed and the paper was retracted in 2013. This misconduct occurred in the clinical trials, which would affect the treatment strategy in many patients. Thus, this misconduct is more serious than that in the STAP cells affair because the fallacious result would harm humans, whereas the STAP cells study was in a basic science. However, this misconduct seems to be perceived less seriously than the STAP cells affair.

Accuracy of Twitter is not always guaranteed. The medical and scientific accuracy of the tweets in this study was questionable for 8.9% of the cases. Accuracy is a constant issue not only in social media but also in Web-based information in general. Caution is always required when using Twitter and other Web-based sites to identify the wrong information. The Japan Internet Medical Association has issued a guide on using Web-based medical information [36]. This guide states that when using medical or health-related information taken from the Internet, members of the public should check that the source is clearly named, that it is backed up by objective evidence, and that the information has been provided by a public medical facility or research institution. If the provider of the information checks the originator or the identity of the account retweeting the information, it may be possible to evaluate its objectivity on the basis of factors such as links included in tweets. To a certain extent, judging the accuracy of information on Twitter is feasible.

The greatest advantage of using Twitter for scientific discussion is the rapidity with which the debate proceeds. However, Twitter also contains inaccurate information and excessive arguments that may become abusive. The latter can have a restraining effect on researchers. Depending on how the functions of Twitter are used, it may be possible to distinguish inaccurate information and to avoid abuse to some extent. Hence, the benefits of the rapid discussion enabled by Twitter, as shown in this study, can be enjoyed while limiting the risks of its disadvantages.

### Limitations

This study showed the advantages of carrying out a scientific discussion on Twitter, but the scope of the study was limited. First, only top tweets were analyzed and an analysis of all tweets might have revealed different views. Second, the content of the discussion on Twitter, particularly on whether a discussion on misconduct in the STAP cells study took place, was not scrutinized. Third, other forms of media such as blogs, weekly magazines, and television were not investigated. Inaccurate images and articles broadcast by other media may also have had an effect on Twitter. Moreover, we did not analyze the accuracy of newspapers. The accuracy of information in newspapers was not necessarily better than that of Twitter. It is possible that the newspaper articles contained inaccurate statements.

The discussion of misconduct in the STAP cells study might have spread rapidly as it involved copying and pasting, a careless behavior familiar to the public. Specialized and complicated misconduct would be less likely discussed by the public on Twitter.

### Conclusions

The discussion on scientific misconduct involving the STAP cell study took place at an earlier stage on Twitter than in newspapers, representatives of the traditional media. Results of the study suggest that the Twitter debate might have contributed to the resolution of the STAP cell scandal.

## Abbreviations

STAP: Stimulus‑Triggered Acquisition of Pluripotency
